# GLP-1 receptor agonists as an adjunct to bariatric surgery for weight loss and metabolic outcome improvement: a systematic review and meta-analysis

**DOI:** 10.1007/s00423-025-03831-4

**Published:** 2025-10-10

**Authors:** Yee Wen Tan, Mengge Shang, Sean Davis, Sivakumar Gananadha

**Affiliations:** 1 Department of Surgery, Canberra Hospital, Canberra, Australia; 2 School of Medicine and Psychology, Australian National University, Canberra, Australia; 3 Department of General Surgery, Canberra Hospital, Yamba Drive, Garran, ACT 2605 Australia

**Keywords:** Bariatric surgery, GLP-1 RA, Weight regain, Weight loss

## Abstract

**Abstract:**

Glucagon-Like- Peptide-1 (GLP-1) receptor agonist has an emerging role in obesity management. This meta-analysis and systematic review evaluated the effectiveness of GLP-1 receptor agonists in aiding weight loss and enhancing cardiometabolic health in patients with insufficient weight loss (IWL) or weight regain (WR) after bariatric surgery.

**Methods:**

A comprehensive literature search (PubMed, Medline, Embase, Cochrane) was conducted following PRISMA guidelines. Included studies involved adults (≥18 years) treated with GLP-1 agonists either before or after bariatric surgery. Primary outcomes assessed were weight and BMI changes; secondary outcomes included metabolic improvements and adverse effects.

**Results:**

19 studies were included in the systematic review and meta-analysis. All included study involves at least one type of GLP-1 agonist for IWL or WR after bariatric surgery with duration of intervention between 3 months to 24 months. All studies showed significant weight and BMI changes from baseline after initiation of different types of GLP-1 agonist, with effects proportionate to length of intervention. Semaglutide outperformed liraglutide in achieving ≥10% and ≥15% weight loss post-surgery. Tirzepatide, a newer GLP-1/GIP agonist, showed even greater weight loss compared to semaglutide over 6 months. A systematic review of 6 studies on the metabolic effects of GLP-1 receptor agonists (RA) post-bariatric surgery highlighted significant improvements in glycemic control, blood pressure, cholesterol levels, and liver function. Adverse effects were mostly mild gastrointestinal symptoms with no severe events reported.

**Conclusion:**

GLP-1 agonists have emerged as a promising alternative to revisional surgery for patients experiencing insufficient weight loss (IWL) or weight regain (WR) after bariatric surgery. Tirzepatide, the newest GLP-1 /GIP agonist, has shown superior results compared to liraglutide and semaglutide. However, more long-term randomized controlled trials are needed to confirm these findings and further assess its effectiveness. Despite this, GLP-1 agonists consistently demonstrate significant weight loss and cardiometabolic improvements when compared to placebo or lifestyle modifications, making them a valuable treatment option for post-bariatric surgery patients.

**Supplementary Information:**

The online version contains supplementary material available at 10.1007/s00423-025-03831-4.

## Introduction

Glucagon-Like Peptide-1 receptor agonists (GLP-1 RA) have emerged as a promising treatment for obesity and related comorbidities [1]. These agonists have been used in the management of diabetes mellitus and obesity, as well as to slow the progression of chronic kidney disease in diabetic patients and improve cardiovascular risk profiles [2, 3]. GLP-1 stimulates glucose-dependent insulin release from the pancreatic islets while reducing plasma glucagon levels, aiding in glucose homeostasis [4]. Additionally, GLP-1 delays gastric emptying, therefore increasing satiety and reducing food intake [4].

A limitation of the natural GLP-1 hormone is its short half-life due to rapid degradation by dipeptidyl peptidase-4 (DPP-4) and high renal clearance. To overcome this, GLP-1 receptor agonists such as liraglutide, semaglutide, and tirzepatide act by reducing DPP-4 binding and minimizing renal clearance, thereby maximizing their effects on weight loss and glycaemic control [5].

While bariatric surgery is the most effective treatment for obesity, some patients experience inadequate weight loss or regain weight post-surgery, often leading to the recurrence of obesity-related comorbidities. GLP-1 analogues have been used as adjunct therapies to bariatric surgery, either preoperatively or postoperatively. Liraglutide and semaglutide have demonstrated 15–17% weight loss, along with cardiometabolic protective properties [6]. Additionally, newer dual GLP-1/GIP receptor agonists, such as tirzepatide, have shown up to 22% weight loss in recent phase 3 trials [7].

The aim of this meta-analysis and systematic review is to evaluate the efficacy of GLP-1 receptor agonists used as adjuncts to bariatric surgery in patients with inadequate weight loss or weight regain, and their effects on obesity-related comorbidities.

## Methodology

**Fig. 1:**
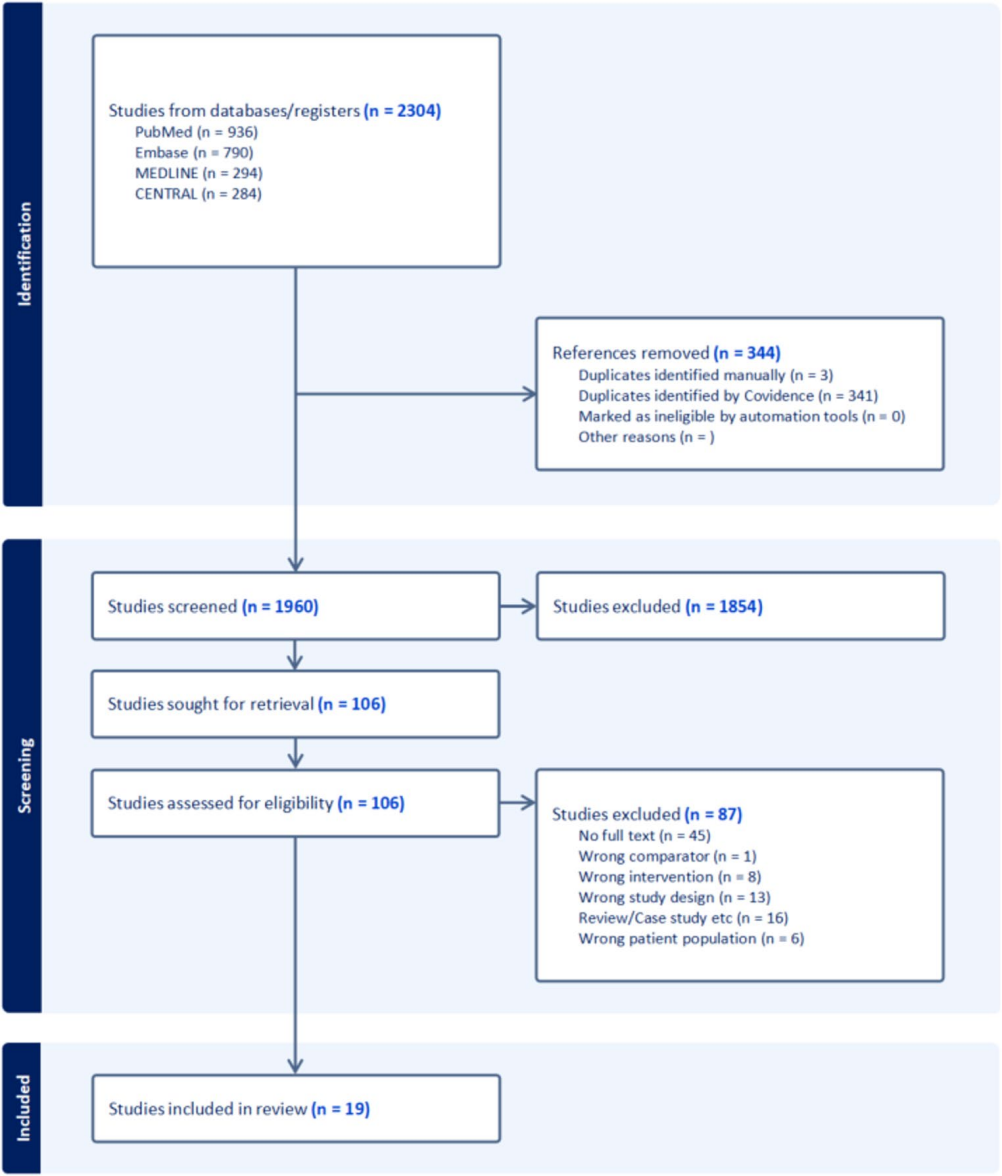
PRISMA Flowchart

The Preferred Reporting Items for Systematic Reviews and Meta-Analyses (PRISMA) criteria were adapted (Fig. 1).

### Literature search strategy

A comprehensive electronic literature search of medical and health science databases was conducted from November 2023 to February 2025 including Ovid MEDLINE, PubMed, EMBASE, and the Cochrane Library. A grey literature search was also performed using Google Scholar. In addition, a manual search of relevant journals and bibliographies of potential articles was conducted to identify additional references. The search was restricted to articles in English and studies involving humans.

The following Boolean search terms were used:

- (Bariatric Surgery) OR (Biliopancreatic Diversion) OR (Roux-en-Y gastric bypass) OR (Gastric banding) OR (weight loss surgery) OR (Biliopancreatic diversion with duodenal switch) OR (Sleeve gastrectomy).
  AND
- (Glucagon-Like Peptide 1 Agonists) OR (Glucagon-Like Peptide 1 Analog) OR (Semaglutide) OR (Exenatide) OR (Liraglutide) OR (Albiglutide) OR (Lixisenatide) OR (Dulaglutide) OR (Tirzepatide).

### Inclusion and exclusion criteria

Due to the limited number of studies available on the topic, we included not only randomized controlled trials (RCTs) but also case-control, cohort, and demographic studies that met the selection criteria. The inclusion criteria involved studies of adult patients (aged 18 years or older) who received a GLP-1 RA either before or after bariatric surgery. We also included the dual GLP-1RA/GIP agonist. Studies not published in English or containing only abstracts were excluded. Additionally, studies on conversional bariatric surgery or endoscopic bariatric procedures were excluded.

### Data extraction

Two authors (YWT and MS) independently selected studies based on the inclusion and exclusion criteria. In cases of disagreement, a third author (SD) resolved the issue. Screening was performed using Covidence (Covidence systematic review software, Veritas Health Innovation, Melbourne, Australia) and data were extracted into a pre-formed Excel file (Microsoft Corporation, Redmond, Washington, USA). The extracted data included the first author, publication year, country, study design, patient demographics, type of GLP-1 RA, dosage, duration of intervention, and various postoperative outcome measures (outlined below).

### Quality assessment of retrieved articles

Quality assessment was performed by two authors (YWT and MS) using Cochrane Collaboration’s tool for assessing risk of bias in randomized trials (RoB 2), which categorizes each RCT as low, some concerns, or high risk for bias in 5 domains: selection, performance, detection, attrition, and reporting biases [8]. For non-randomized studies, the Risk Of Bias In Non-randomized Studies of Interventions (ROBINS-I) was used which evaluated key points such as selection of participants, baseline differences between groups, and incomplete data outcome [9]. [Refer to supplementary material]

### Outcome measures

The primary outcomes included total weight loss, excess weight loss, and BMI reduction with adjunct GLP-1 treatment either before or after bariatric surgery. Secondary outcomes included metabolic changes including blood pressure, HbA1c, and cholesterol levels. Another secondary outcome measure is the adverse reactions related to GLP-1 RA.

### Statistical analysis

All statistical analysis was performed using Review Manager [RevMan], Version 5.4.1 (The Cochrane Collaboration,2020). Odds ratios (OR) were used to summarise dichotomous data, and mean difference (MD) were used for continuous data. If means and standard deviations were not reported, they were calculated from the data available using methods described by Hozo et al. [10] Pooled analysis was performed comparing GLP-1 RA to baseline as well individual GLP-1 RA to calculate Odds Ratios (OR) and 95% confidence intervals (CI). I^2^ statistics with p-value set to *p* < 0.10 for significance, were utilised to assess heterogeneity. I^2^ scores > 50% denoted significant heterogeneity. When the heterogeneity test was statistically significant, a random effects model was used which assumes variation in treatment effects between studies and estimates a more conservative overall treatment effect with wider confidence intervals. Where heterogeneity was not significant, a fixed effects model was used.

## Results

Our initial literature search yielded 2,304 studies, of which 344 were identified as duplicates and subsequently removed, leaving 1,960 articles for screening. After an initial review of titles and abstracts, 1,854 studies were deemed irrelevant and excluded. The remaining 106 studies were further analysed in detail. Ultimately, 19 studies that included the use of GLP-1 agonist either before or after bariatric surgery, focusing on outcomes related to weight or BMI changes and metabolic changes, were included in the final review [7, 11–26]. 

Of the 19 studies included, 3 studies were Randomised controlled studies, 13 studies were retrospective cohort studies, 1 was prospective cohort studies, and 2 retrospective observational studies. Each study involved at least one type of GLP-1 RA administered either before or after bariatric surgery, with intervention durations ranging from 3 weeks to 21 months. The GLP-1 RA used across these studies included liraglutide, semaglutide, dulaglutide, exenatide, and tirzepatide, in various dosages and preparations. The baseline characteristics of all studies are presented in Table 1.

**Table 1:** Baseline characteristics of included studies

Study	Study type	Total patients	Time of initiation of GLP-1 agonist	Inclusion	GLP-1 RA	Duration of GLP-1 RA treatment	Number	BMI * (Kg/m2)	Weight * (Kg)	Number	BMI * (Kg/m2)	Weight* (Kg)	Total % weight loss	Follow up (months)	Number	BMI * (Kg/m2)	Weight* (Kg)	Total % weight loss	Follow up (months)
Jensen 2023	R Obs	50	72 months	WR	Liraglutide 3mg daily	6 months				29			7.3 (1.8)	6					
					Semaglutide 1mg weekly	6 months				21			9.8 (1.2)	6					
					Total group		50	34 (1.75)	90.5 (6.12)	50	31.5 (1.92)	83.1 (5.45)	8.8 (1.55)	6					
Murvelashvili 2023	R Obs	207	8.24 years	WR	Liraglutide 3mg daily	>3 months 12 months	92		114.4 (27.56)	92			8.77 (6.04)	12					
			7.8 years		Semaglutide 1mg weekly	>3 months 12 months	115		110.7 (24.5)	115			12.92 (6.4)	12					
			8 years		Total group		207		112.31 (25.88)					12					
Jamal 2024	R Cohort	115	71.8 (51.1) months *	WR	Semaglutide Increasing dose	>3 months 12 months	70	33.9 (6)	90.1 (19.4)	70	31.9 (6)	84.9 (19.3)		3	70	30.4 (6)	81 (19)		6
					Tirzepetide Increasing dose	>3 months 12 months	45	36.9 (7.1)	100.2 (28.5)	45	33.5 (6.8)	91.2 (27.3)		3	45	32.1 (6.9)	87.6 (28.3)		6
					Total group		115	35.1 (6.0)	94 (23.8)	115	32.5 (6.4)	87.3 (22.9)		3	115	31.1 (6.4)	83.2 (22.7)		6
Lautenbach 2022	R Cohort	44	64.7 (6.4) months*	WR, IWL	Semaglutide Increasing dose	3–6 months	44	38.3 (6.4)	113.5 (25.2)	38	36 (6.1)	106.5 (24.5)	6 (4.3)	3	20	36.2 (6.7)	105.7 (25.1)	10.3 (5.5)	6
Lautenbach 2023	R Cohort	29	64.5 (51.9) months*	WR, IWL	Semaglutide increasing dose	12 months	29	38.3 (6.1)	110.8 (22.2)	29	34.5 (6.3)	99.2 (22.9)	11.2 (5.6)	6	29	33.0(7.2)	95.4 (24.0)	14.7 (8.9)	12
Elhag 2022	R Cohort	119	56.47 (32.39) months*	WR, IWL	Liraglutide increasing dose	6–12 months	107	37.61 (6.42)	96.75 (18.65)	107	35.37 (5.89)	91.01 (17.30)	5.97 (5.71)	6	110	34.42 (4.82)	88.44 (15.47)	6.93 (7-21)	12
		26	42.54 (26.05) months*				25	38.36(9.77)	101.76 (31.1)	25	36 (10.29)	95.68 (33.29)	6.41 (7.11)	6	25	36.71 (9.39)	97.65 (32.13)	4.99 (8.15)	12
		145	54.1 (31.75) months*				145												
Colbourne 2023	P Cohort	68	>12 months	WR, IWL	Liraglutide increasing dose	>3 months	68	35.2 (2.42)	95.8 (6.05)	68	32.3 (2.47)	90.3 (8.66)	5.30 (1.53)	16					
Jamal 2023	R Cohort	57		WR, IWL	Liraglutide increasing dose	3 months	57		96.12 (29.26)	57		90.19 (26.82)	6.2 (6.0)	3					
Muratori 2022	R Cohort	62	70.7 (43.7) months	WR, IWL	Liraglutide increasing dose	3–18 months	62	34.2 (4.8)		62	28.04 (3.66)			10.44					
Rubio 2021	R Cohort	23		WR,IWL	Liraglutide daily	12 months	12				30.5 (4.7)			12					
					Liraglutide alternate days	6 + 6 months	11				29.7 (3.3)			12					
					Liraglutide total		23	36.6 (4.6)			30.1 (4)			12					
Rye 2018	R Cohort	20	73.3 (72.9) months	WR,IWL	Liraglutide increasing dose	7 months	20		117.91 (27.95)	20		108.87 (28.10)		4	20		105.59 (27.49)		7
Gorgojo-Martinez 2016	R cohort	15	5.2 years	WR, IWL, DM	Liraglutide increasing dose	24 months	15		106 (27.88)	15		102.6 (26.72)		24					
Pajecki 2016	R cohort	15	5.6 years	WR, IWL	Liraglutide increasing dose	2–7 months	15		100.9 (18.3)	15		93.5 (17.4)		4.2					
Wharton 2019	R Cohort	117	8 years	WR, IWL	Liraglutide increasing dose	variable- mo minimum	117	42.5 (9.6)		117			5.5 (6.2)	7.6 (7.1)					
Vinciguerra 2024	R Cohort	119		Wr,IWL	Liraglutide increasing dose	3–6 months	119	37.6 (5.3)	100.9 (17.2)	119	35.6 (5.3)	95.3 (16.8)		3	119	34.2 (5.2)	91.5 (16.49)		6
Horber 2021	R Cohort	34	6 years	WR, IWL	Liraglutide increasing dose	24 months	34	31.2 (4)	84 (13)	34	26.4 (3.5)	72 (9)		24					
Thakur 2021	RCT	23	6 weeks		Liraglutide increasing dose	6 months	12	36.5 (5.2)	103.5 (20.3)	12	34 (4.4)	94.2 (17.6)	20.6 (6.3)	12	12	30.9 (4)	85.1 (13.5)	28.2 (5.7)	24
					Placebo		11	37 (3.9)	92 (32.4)	11	34.5 (3.5)	84.8 (11.4)	17.7 (6.1)	12	11	32.1 (3)	79.2 (10.6)	23.2 (6.2)	24
Miras 2019	RCT		3.8 years		Liraglutide increasing dose	6 months	53	36.1 (7.8)	100.7 (20.7)					6.5					
			3.8 years		Placebo		27	37 (7.7)	103.5 (27)					6.5					
Mok 2023	RCT	35	55.1 months		Liraglutide increasing dose	6 months	35	41.6 (6.9)	116. (23.6)										
		35	49.1 months		Placebo		35	44.6 (8.3)	123.5 (24.8)										

*R cohort*, Retrospective cohort study, *R obs*, Retrospective Observational study, *RCT*, Randomised Control Study

*Data are reported as mean +/- SD

## Effect of GLP-1 RA on primary outcomes

**Fig. 2:**
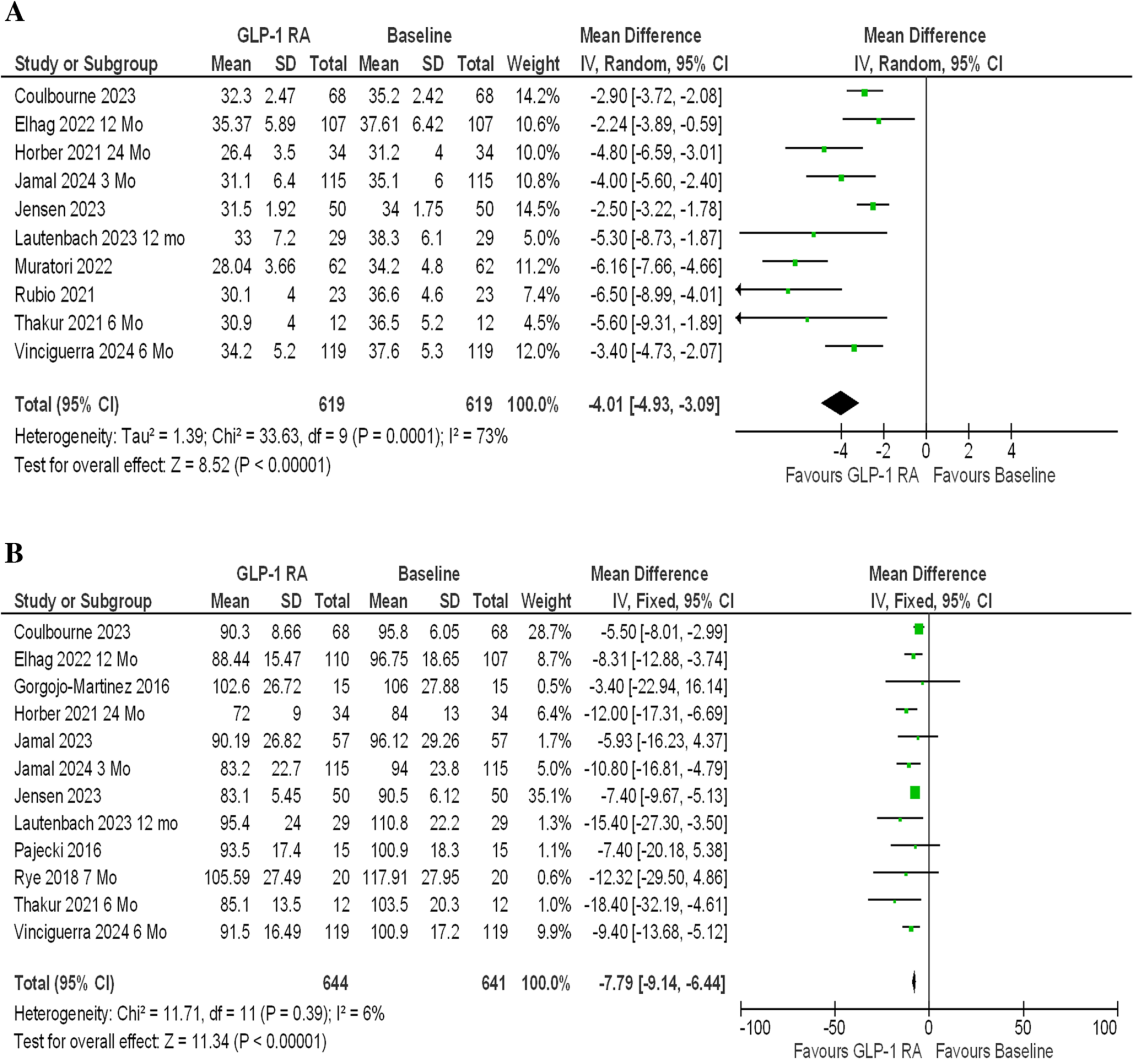
(**A**) Any GLP-1 RA- longest follow up- BMI Change. (**B**) Any GLP-1 RA- longest follow up- Weight Change

10 studies which includes any GLP-1 agonists were included in further meta-analysis, shows statistically significant improvement in BMI [MD-3.37 (95% CI, −2.78 to −2.95); *P* < 0.0001; *I*^2^ = 73%; Fig. 2 A] and weight loss [MD – 7.79 kg (95% CI, −9.14 to −6.44); *P* < 0.0001], *I*^2^ = 6%; Fig. 2B] from baseline [7, 11, 13, 16–18, 20, 21, 26, 27]. High heterogenicity were detected in for BMI changes [figure 1 *Refer to supplementary data for funnel plot graphs].

**Fig. 3:**
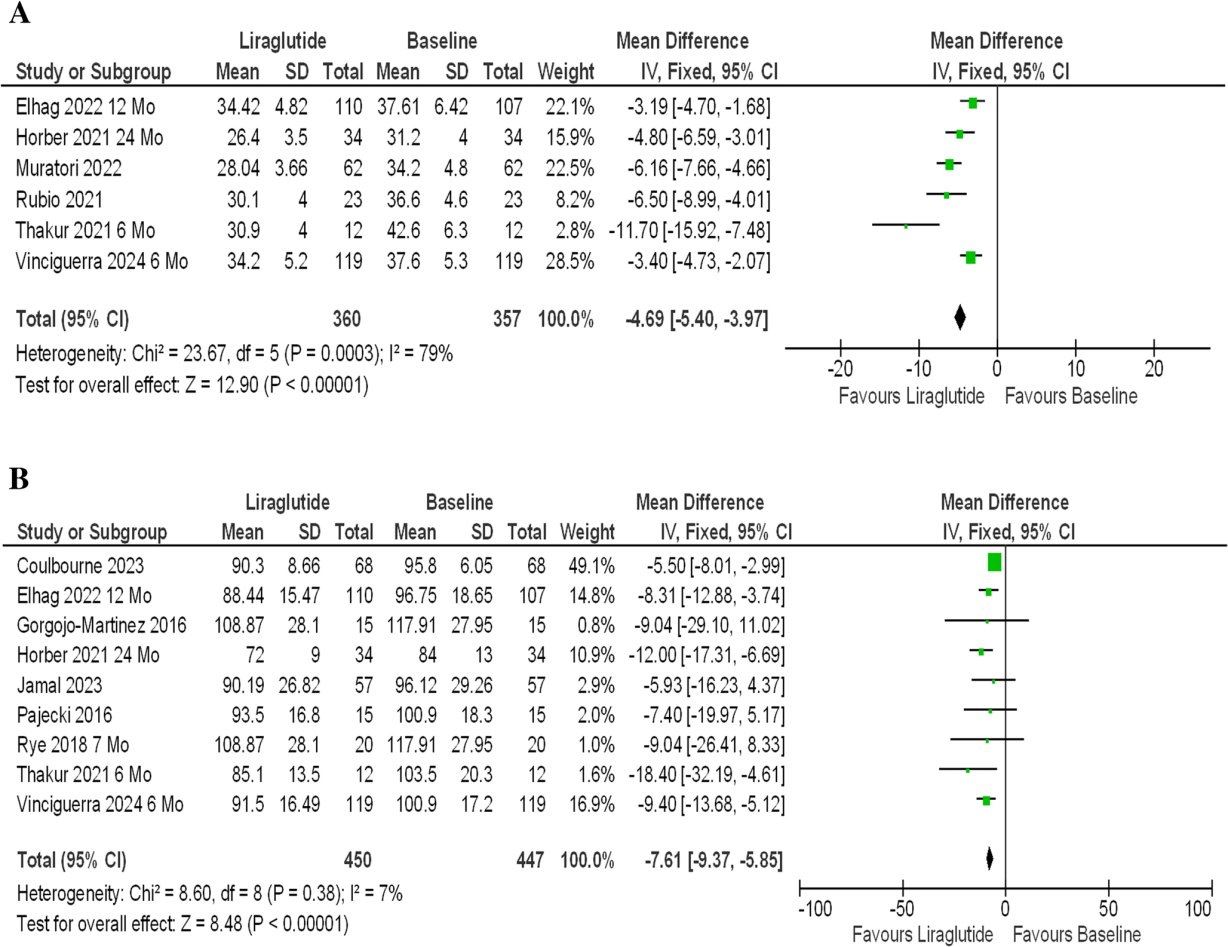
(**A**) Liraglutide - longest follow up- BMI Change. (**B**) Liraglutide - longest follow up- Weight Change

**Fig. 4:**
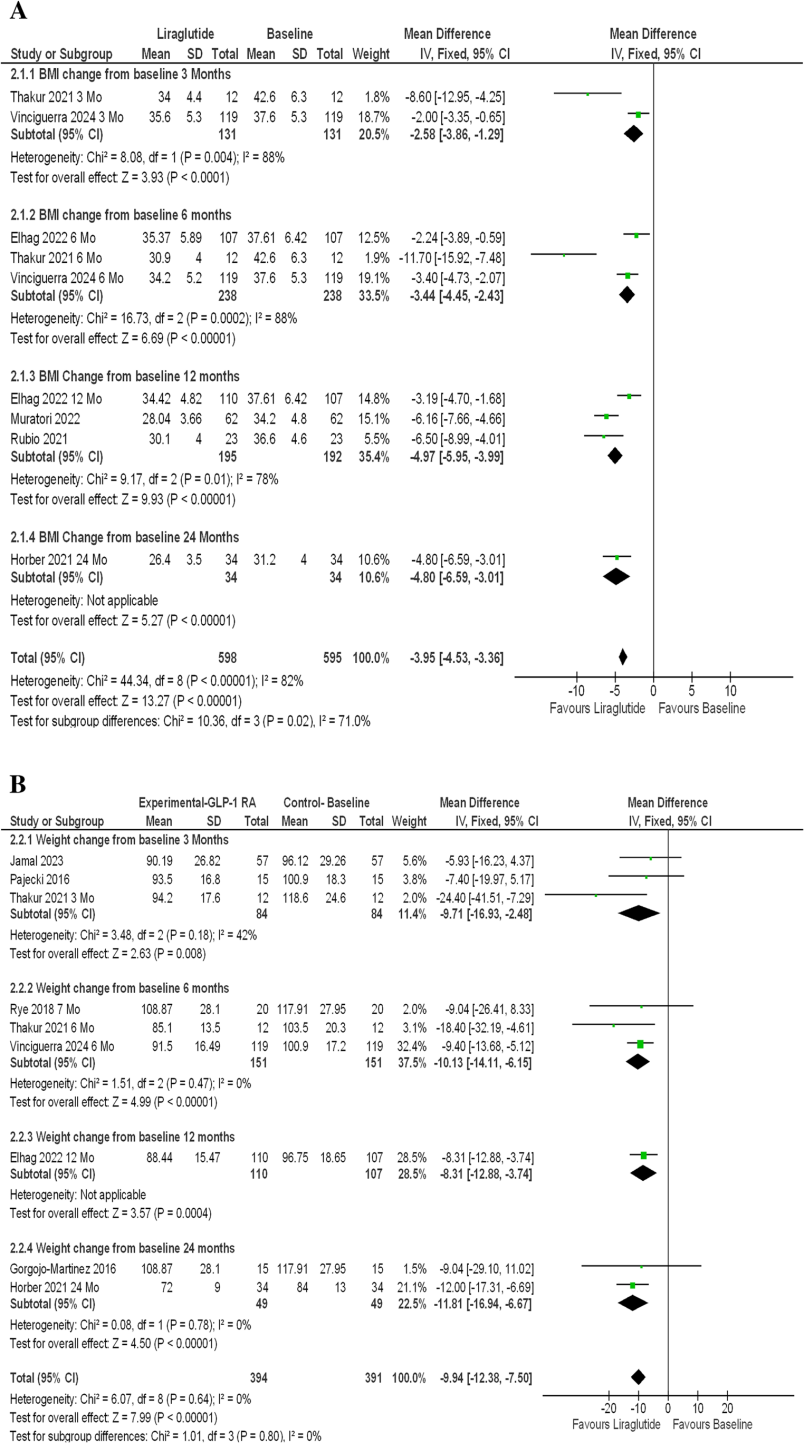
(**A**) Liraglutide- BMI change from baseline subgroup analysis. (**B**) Liraglutide- Weight change from baseline subgroup analysis

**Fig. 5:**
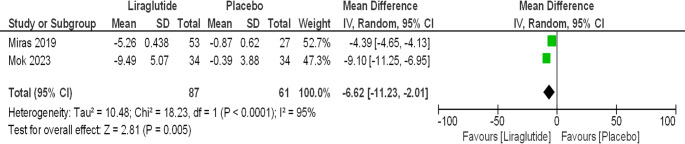
A Liraglutide vs. placebo. Total weight loss

Liraglutide being the first GLP-1 agonist approved for weight management and longest in the market were used in majority of the studies. 6 studies that involve liraglutide showed statistically significant improvement in both BMI changes [MD-4.69 (95% CI, −5.40 to −3.97); *P* < 0.00001; *I*^2^ = 79%; Fig. 3 A] and weight loss [MD −7.61 kg (95% CI, −9.37 to −5.85); *P* < 0.0001; *I*^2^ = 7%; Fig. 3B]. Further subgroup analysis were performed to evaluate the impact of duration of intervention on degree of weight loss and BMI change in these studies involving Liraglutide, showing a directly proportional relationship between the two [Figure 4 A and B]. Weight loss of [MD −9.71 kg (95% CI, −16.93 to −2.48); *P* = 0.008; *I*^2^ = 42%] at 3 months, [MD −10.31 kg (95% CI, −14.11 to −6.15); *P* < 0.00001; *I*^2^ = 0%] at 6 months, [MD −8.31 kg (95% CI, −12.88 to −3.74); *P* = 0.0004] at 12 months and [MD – 11.81 kg (95% CI, −16.94 to −6.67); *P* < 0.00001; *I*^2^ = 0%] at 24 months were reported. In total, studies using liraglutide reported weight loss of nearly 10 kg with consistent and statistically significant results across all studies [MD −9.94 kg (95% CI, −12.38 to −7.50); P, 0.00001; *I*^2^ = 0%; Fig. 4B]. Similar findings were observed in analysis of Liraglutide group vs. placebo group with weight loss of [MD – 6.62 kg (95% CI, − 11.23 to −2.01); *P* < 0.0001; *I*^2^ = 95%; Fig. 5].

### Semaglutide

**Fig. 6:**
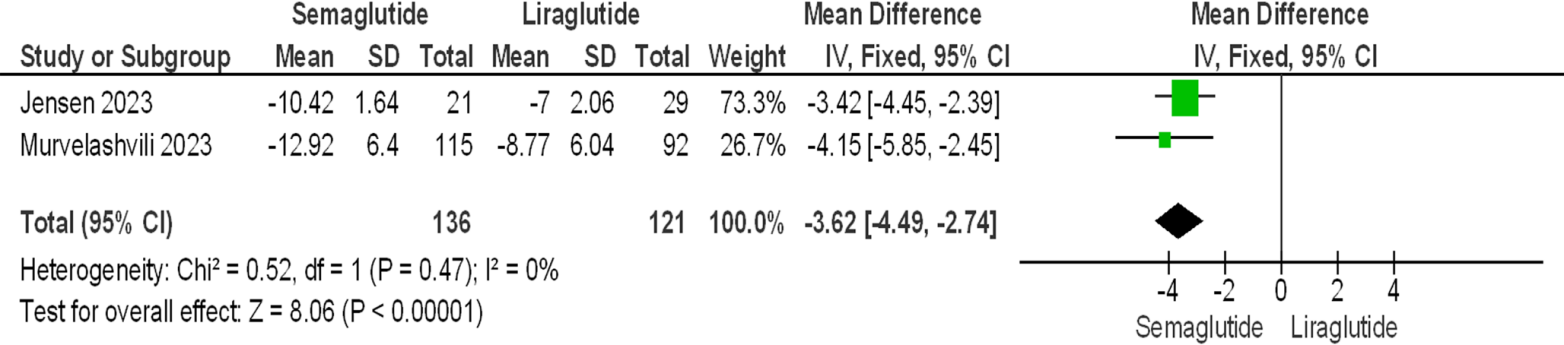
Semaglutide vs. Liraglutide. Percentage weight loss

Semaglutide was found to show superior results in weight loss as compared to liraglutide in studies done by Jensen et al. and Murvelashvili et al. with mean difference of [−3.62 kg (95% CI, −4.49 to 2.74 kg); *P* < 0.00001; *I*^2^ = 0%; Figure 6] [26, 28]. Study by Murvelashvili et al. [28], which analysed weight loss among 207 post-bariatric surgery patients who received either subcutaneous semaglutide 1 mg weekly (*n* = 115) or subcutaneous liraglutide 3.0 mg daily (*n* = 92), found that patients in the semaglutide group had nearly twice the odds of losing ≥ 10% and ≥ 15% of their body weight compared to the liraglutide group. The adjusted odds ratios (aOR) were 2.34 (95% CI: 1.28 to 4.29) and 2.55 (95% CI: 1.22 to 5.36), respectively. The study, with a longer intervention period of 12 months, reported significant weight loss of 12.92% (95% CI: 14.09–11.75%) in the semaglutide group (*p* < 0.001) compared to 8.77% (95% CI: 7.54–0.01%) in the liraglutide group (*p* < 0.001).

#### Tirzepartide

Tirzepartide was found to be the most effective among all agents however there was only one study available with adequate data for meta-analysis. The only study involving Tirzepartide was done by Jamal et al. [27] with total 115 participants received either S/C semaglutide starting at 0.25 mg weekly or S/C Tirzepartide 2.5 mg weekly at 71.8 (51.1) months after sleeve gastrectomy for 6 months. Weight loss from Tirzepartide group was evidently greater than semaglutide group at both 3rd and 6th months, [6.0 (3.6)% and 10.3 (5.9)%, *p* < 0.05 for semaglutide group vs. 9.3 (4.3)% and 15.5 (6.3)%, *p* < 0.05 for Tirzepatide group]

#### Non responders

Non-responders to GLP-1 receptor agonist adjunct therapy (defined as achieving < 5% total weight loss) following bariatric surgery were most prevalent among patients treated with liraglutide (27.12%), followed by semaglutide (19.1%), and tirzepatide (2.9%). These findings align with the comparative effectiveness of each agent in promoting weight loss and BMI reduction, as previously discussed (Table 2).

**Table 2:** Number of non-responders to GLP-1 receptor agonist and duration of intervention

Study	Number	Duration	GLA-1 RA	Non responders
				*n* (%)
Colbourne et al. 2023	68	3 months	Liraglutide	7/68 (10.3)
Elhag et al. 2022	110	12 months	Liraglutide	44/110 (46)
Vinciguerra et al. 2024	107	6 months	Liraglutide	7/107 (6)
Jamal et al. 2023	57	3 months	Liraglutide	26/57 (45.6)
Rye et al. 2018	20	7 months	Liraglutide	2/20 (10)
Pajecki et al. 2012	15	3 months	Liraglutide	3/15 (20)
Horber et al. 2021	34	24 months	Liraglutide	3/34 (9)
Miras et al. 2019	48	6 months	Liraglutide	26/48 (54)
Mok et al. 2023	32	6 months	Liraglutide	9/32 (28.1)
Jensen et al. 2023	29	6 months	Liraglutide	9/29 (31)
Murvelashvili et al. 2022	92	12 months	Liraglutide	30/92 (32.6)
				**166/612 (27.12%)**
Lautenbach et al. 2023	29	12 months	Semaglutide	2/29 (10.3)
Jensen et al. 2023	21	6 months	Semaglutide	3/21 (14.2)
Murvelashvili et al. 2022	115	12 months	Semaglutide	26/115 (22.6)
Jamal et al. 2024	70	6 months	Semaglutide	14/70 (20)
				**45/235 (19.1%)**
Jamal et al. 2024	35	6 months	Tirzepatide	1/35 (2.9)

Data are presented in mean (standard deviation)

Abbreviations: *FBG* Fasting Blood Glucose, *SBP *Systolic Blood Pressure, *DBP *Diastolic Blood Pressure, *HDL *High Density Lipoprotein, *LDL *Low Density Lipoprotein

## Secondary outcomes

Due to the heterogeneity and non-uniform reporting of secondary outcomes, a meta-analysis was not feasible. Therefore, a systematic review was conducted to synthesize findings on changes in metabolic outcomes of glycaemic control, blood pressure, and cholesterol levels with introduction of GLP-1 agonists.

A study by Lautenbach et al. demonstrated significant reductions in HbA1c levels (from 5.4% ± 0.4 to 5.1% ± 0.4, *p* < 0.001) and complete resolution of pre-diabetes in all patients (*p* < 0.05) treated with semaglutide post-bariatric surgery. Triglyceride levels also showed a significant decrease (*p* = 0.016), while reductions in total cholesterol and LDL levels were observed, though they were not statistically significant (*p* = 0.52, *p* = 0.727, respectively) [11]. 

Similar improvement in HbA1c were seen in a study by Miras et al., where the treatment effect from baseline to 26 weeks was − 1.22 (95% CI: −1.80 to −0.64, *p* = 0.0001) [14]. A study by Vinciguerra further supported these findings, showing significant improvements in both systolic and diastolic blood pressure (*p* < 0.0001), as well as triglyceride levels (*p* = 0.0001), with subcutaneous liraglutide starting at 0.6 mg daily and titrated by 0.6 mg increments to the minimum effective dose for 24 weeks. The mean dosage of liraglutide in this study was 2.4 mg. Further details of baseline and post-GLP-1 agonist metabolic parameters are presented in Table 3 [16].

**Table 3:** Secondary outcomes in term of metabolic change post introduction of GLP-1 agonists

Study	Lautenbach 2022	Elhag 2022	Mok 2023	Jensen 2023	Thakur 2021	Miras 2019
Intervention	Semaglutide Increasing dose	Liraglutide increasing dose	Liraglutide increasing dose	Liraglutide 3 mg daily	Liraglutide increasing dose	Liraglutide increasing dose
				Semaglutide 1 mg weekly		
Duration of intervention	3 to 6 months	6 to 12 months	6 months	6 months	6 months	6 months
Hba1c Pre-treatment (%)	5.3 (0.4)	5.95 (1.00)	5.8 (0.7)	7.2 (6.6, 8.0)	6.0 ± 1.5	7.9 (1.39)
Hba1c Post treatment (%)	5.2 (0.2)	5.95 (1.62)	5.53 (0.79)	6.4 (6.3, 6.6)	7·6 (1·65)	7.6 (1.44)
FBG Pre-treatment (mmol/L)		5.63 (1.66)			5.92 +/- 2.13	
FBG post treatment (mmol/L)		5.47 (1.25)			5.47 +/- 1.89	
SBP Pre-treatment (mmHg)	125.9 (10.8)	121.11(16.23)	131.3 (15.0)			
SBP post treatment (mmHg)	119.2 (7.6)	117.5 (15.17)	125.0 (22.2)			
DBP Pre-treatment (mmHg)	80.5 (7.6)	73.58 (9.99)	75.9 (10.4)			
DBP post treatment (mmHg)	75.6 (5.2)	72.51 (10.78)	75.5(16.8)			
Total Cholesterol Pre-treatment (mmol/L)	4.72 (0.92)	4.78 (0.89)	5.3 (1.2)			
HDL Pre-treatment (mmol/L)	1.43 (0.39	1.61 (0.42)	1.4 (0.4)			
LDL Pre-treatment (mmol/L)	2.47 (0.80)	2.69 (0.86)	3.3 (0.9)			
Total Cholesterol Post treatment (mmol/L)	4.4(0.74)	4.79 (0.99)	4.83 (1.33)			
HDL Post treatment (mmol/L)	1.30(0.43)	1.53 (0.33)	1.30 (0.44)			
LDL Post treatment (mmol/L)	2.31 (0.72)	2.82 (0.86)	3.04(1.07)			

## Adverse effect

Adverse effects of liraglutide were reported in 7 studies included in this review, with gastrointestinal side effects being the most common [14, 15, 18, 19, 22, 23, 26]. However, no serious adverse effects were directly attributed to GLP-1 agonists. (Table 4)

**Table 4:** Total number of adverse events secondary to GLP-1 agonists

	Total patients = 516
Adverse event	n (%)
Decreased appetite	15 (2.91)
Dizziness	8 (1.55)
Taste dysgeusia	9 (1.74)
Dry Mouth	6 (1.16)
Fatigue	9 (1.74)
Headache	21 (4.07)
Peripheral oedema	1 (0.19)
Hypogylcemia	3 (0.58)
Injection-site bruising	5 (0.97)
Rash	5 (0.97)
Sweating	2 (0.39)
Back pain	2 (0.39)
Depression	1 (0.19)
Hot flushes	1 (0.19)
Flu-like symptoms	4 (0.78)
Palpitation	3 (0.58)
Urinary tract infection	1 (0.19)
Respiratory infection	7 (1.36)
Cellulitis	1 (0.19)
Progression of chronic kidney disease	2 (0.39)
Lymphoma	1 (0.19)
**Gastrointestinal**	
Nausea	114 (22.09)
Vomiting	19 (3.68)
Diarrhoea	22 (4.26)
Constipation	41 (7.95)
Indigestion/dyspepsia	7 (1.36)
Abdominal discomfort/pain	6 (1.16)
Bloating	5 (0.97)
Gastro-oesophageal reflux	12 (2.33)
Dysphagia	1 (0.19)
Gastroenteritis	1 (0.19)
Pancreatitis	2 (0.39)
Death	Nil

Although the overall incidence of adverse effects was relatively high (ranging from 36% to 80%, they were not the primary reason for participant dropout in any of the studies [14, 15, 23, 26]. The dropout rate averages between 11.25 and 63%. Highest dropout rate of 63% was observed in studies done by Wharton et al. [23] studying 117 patients who had liraglutide post bariatric surgery for inadequate weight loss. Surprisingly the most common reported reason for dropout was lack of effectiveness for weight management, however the duration of intervention of this group of patients was uncertain. Colbourne et al. [18] reported that only 19.5% of the 60.2% dropout rate was due to adverse effects, whereas cost of treatment remains the main reason for drop out. Mok et al. [15] found that liraglutide was potentially better tolerated in patients who had undergone bariatric surgery, with fewer gastrointestinal side effects compared to non-surgical obese populations studied elsewhere.

## Discussion

This meta-analysis and systematic review examined the effectiveness of GLP-1 agonists in managing weight regain (WR) or inadequate weight loss (IWL) following bariatric surgery. The findings suggest that GLP-1 receptor agonists (RAs) are an effective treatment option for patients experiencing IWL or WR post-surgery. Significant weight loss was observed across all three GLP-1 RAs- liraglutide, semaglutide, and tirzepatide. Among these, the dual GLP-1 RA/GIP agonist, tirzepatide, led to the greatest weight loss, followed by semaglutide, and then liraglutide. In terms of inadequate weight loss (defined as less than 5% of total body weight lost), liraglutide had the highest rate of non-responders at 27.1%, followed by semaglutide at 19.1%, and tirzepatide, which was evaluated in only one study, had the lowest rate at 2.7%. 6

Insufficient weight loss (IWL) was defined as an excess weight loss percentage (EWL%) of less than 50% within 18 months post-bariatric surgery. Weight regain (WR), on the other hand, refers to progressive weight gain following initial weight loss success. However, there are no standardized definitions of WR or IWL, and the definitions used in various studies vary widely [29].

Various factors contribute to insufficient weight loss (IWL) and weight regain (WR), including dietary nonadherence, lack of physical activity compliance, mental health disorders, and anatomical issues following surgery. Historically, revisional surgery has been the primary treatment for WR and IWL. However, pharmacotherapy, particularly GLP-1 agonists, has gained increasing attention in recent years due to their beneficial hormonal effects on satiety and hunger regulation [30, 31]. 

GLP-1 agonists, originally developed as anti-diabetic agents, have shown significant benefits beyond glycemic control, including substantial weight loss, metabolic improvements, and cardiovascular protection. Liraglutide, semaglutide, and tirzepatide have become effective alternatives to revisional surgery for patients experiencing weight regain (WR) or insufficient weight loss (IWL), with weight loss ranging from 5.3 to 29.1% [32]. Liraglutide, introduced in 2010, demonstrating significant weight reduction compared to placebo [13–15]. Semaglutide, FDA-approved in 2021 for weight management in patients with type 2 diabetes, has shown superior weight loss outcomes compared to liraglutide [33]. To date, four retrospective studies have demonstrated semaglutide’s superior weight loss effects over liraglutide [11, 12, 26, 28]. Tirzepatide, the newest GLP-1 agonist approved in 2023, has demonstrated even greater efficacy in weight reduction, as seen in several studies comparing it to semaglutide [27]. It is administered as a subcutaneous injection, starting at 2.5 mg every four weeks, with dose increases of 2.5 mg every four weeks, up to a maximum of 15 mg [34].

Studies [26, 28] comparing the weight loss effects of liraglutide vs. semaglutide have shown superior results for the semaglutide group. In the Murvelashvili study, the adjusted odds of losing ≥ 10% and ≥ 15% of body weight were more than two times higher in the semaglutide group compared to the liraglutide group: adjusted odds ratios (aOR) = 2.34 (95% CI: 1.28 to 4.29) for ≥ 10% weight loss, and 2.55 (95% CI: 1.22 to 5.36) for ≥ 15% weight loss. However, these differences were not statistically significant for weight loss ≥ 5% or ≥ 20% [28]. 

The study by Jamal et al. reported clinically significant weight loss in both the tirzepatide and semaglutide groups, with superior results in the tirzepatide group. After 6 months, 97.1% of patients on tirzepatide achieved > 5% weight loss (vs. 80% in the semaglutide group), 74% achieved > 10% weight loss (vs. 48.6% in the semaglutide group), and 57.1% achieved > 15% weight loss (vs. 26% in the semaglutide group) (*p* < 0.001). However, none of the patients in the semaglutide group received a dose higher than 2 mg, the maximum recommended dose for weight loss, which could introduce some bias in the results [13]. 

In contrast, the study by Le Roux et al. demonstrated greater reductions in body weight% in patients on tirzepatide (10 mg and 15 mg) compared to those on semaglutide 2.4 mg. The mean differences in body weight reduction were as follows: tirzepatide 10 mg, −4.67% (95% CI: −5.91% to −3.43%); tirzepatide 15 mg, −5.92% (95% CI: −7.16% to −4.68%)—both with *p* < 0.001 [34]. 

The metabolic benefits of GLP-1 agonists extend beyond weight loss. These drugs enhance insulin secretion, delay gastric emptying, and improve satiety, which contributes to better glycemic control without causing hypoglycemia [12–18, 25, 26, 28]. The incretin effect of GLP-1 agonists reduces endothelial dysfunction caused by non-enzymatic glycation of proteins in a hyperglycemic state. It also affects calcium transport and lipid metabolism, which contributes to the pathophysiology of diabetic cardiomyopathy [35]. 

GLP-1 agonists also exhibit cardioprotective properties, as highlighted by the SELECT trial. This large-scale trial (*n* = 17,604), conducted in the United States, investigated the cardioprotective effects of semaglutide 2.4 mg compared to placebo. The results showed a significant 20% reduction in the risk of three-point major cardiovascular events (MACEs)—including cardiovascular death, non-fatal myocardial infarction, and non-fatal stroke (hazard ratio [HR] 0.80, 95% CI: 0.72–0.90; *p* < 0.001) [36]. Additionally, GLP-1 agonists have demonstrated positive effects on blood pressure, lipid profiles, and endothelial function [12, 15, 17].

Gastrointestinal adverse effects, such as nausea, vomiting, gastroparesis, and pancreatitis, were among the most reported side effects of GLP-1 agonists [37]. Nausea was the most frequent adverse effect, and it was found to be dose-dependent, improving with continued treatment. Other reported side effects include non-specific systemic reactions such as allergy, angioedema, rash, injection site reactions, and headaches. Renal injuries have been attributed to acute tubular necrosis resulting from volume depletion secondary to nausea and vomiting, though no direct link to GLP-1 agonists has been demonstrated [37]. 

Animal model studies suggest that GLP-1 may increase pancreatic amylase release, potentially contributing to hyperplastic or neoplastic changes in pancreatic duct glands over time. This was hypothesized to cause obstruction of small pancreatic ductules, leading to delayed-onset acute pancreatitis or even pancreatic cancer. However, multiple meta-analyses have found no significant association between GLP-1 agonists and acute pancreatitis [38, 39].

When examining the adverse effect profiles of different GLP-1 agonists, a study by Rubino et al. reported a higher incidence of adverse events in patients on semaglutide compared to liraglutide (84.1% vs. 82.7%), with the highest incidence occurring during or shortly after dose increments [40]. The adverse effect profiles of tirzepatide and semaglutide were found to be similar at lower doses, but the dropout rates increased at higher doses of tirzepatide (10 mg and 15 mg) compared to 5 mg [41]. Munoz et al. also reported that gastrointestinal side effects were more prevalent during the first month of GLP-1 agonist initiation or during dose titration of liraglutide [42]. 

One of the strengths of this review is its inclusion of the most recent studies including those on tirzepatide, which were not featured in previous systematic reviews. Additionally, the focus on the cardiometabolic benefits of GLP-1 agonists offers a broader perspective on their role, extending beyond weight loss. However, the heterogeneity of the included studies and the lack of consistent data limited the ability to perform a meta-analysis. Furthermore, there is a notable scarcity of randomized controlled trials, particularly for semaglutide and tirzepatide, which would provide more accurate and robust comparisons between different GLP-1 agonists.

Several limitations were identified in this study. Notably, there is significant variability in the study designs and the quality of the studies included. Missing data and differences in the methods used to measure central tendency pose additional challenges for conducting a meta-analysis. As a result, fewer articles qualified for data extraction in the meta-analysis, particularly in the analysis of secondary outcomes. Furthermore, the lack of uniformity in reporting styles complicates any meaningful analysis of secondary outcomes and side effects.

## Conclusions

GLP-1 agonists have emerged as a promising alternative to revisional surgery for patients experiencing insufficient weight loss (IWL) or weight regain (WR) after bariatric surgery. Tirzepatide, the newest GLP-1 agonist has shown superior results compared to liraglutide and semaglutide. However, more long-term randomized controlled trials are needed to confirm these findings and further assess its effectiveness. Despite this, GLP-1 agonists consistently demonstrate significant weight loss and cardiometabolic improvements when compared to placebo or lifestyle modifications, making them a valuable treatment option for post-bariatric surgery patients, especially those with type 2 diabetes.

## Supplementary Information

Below is the link to the electronic supplementary material.

Supplementary Material 1

Supplementary Material 2

## Data Availability

No datasets were generated or analysed during the current study.
