# Supplementary figures and images for: GLP-1 receptor agonists as an adjunct to bariatric surgery for weight loss and metabolic outcome improvement: a systematic review and meta-analysis
Source: Langenbecks Arch Surg. 2025 Oct 10;410(1):295. doi: 10.1007/s00423-025-03831-4 (PMC12513976; doi:10.1007/s00423-025-03831-4)

**Quality Assessment of included studies**


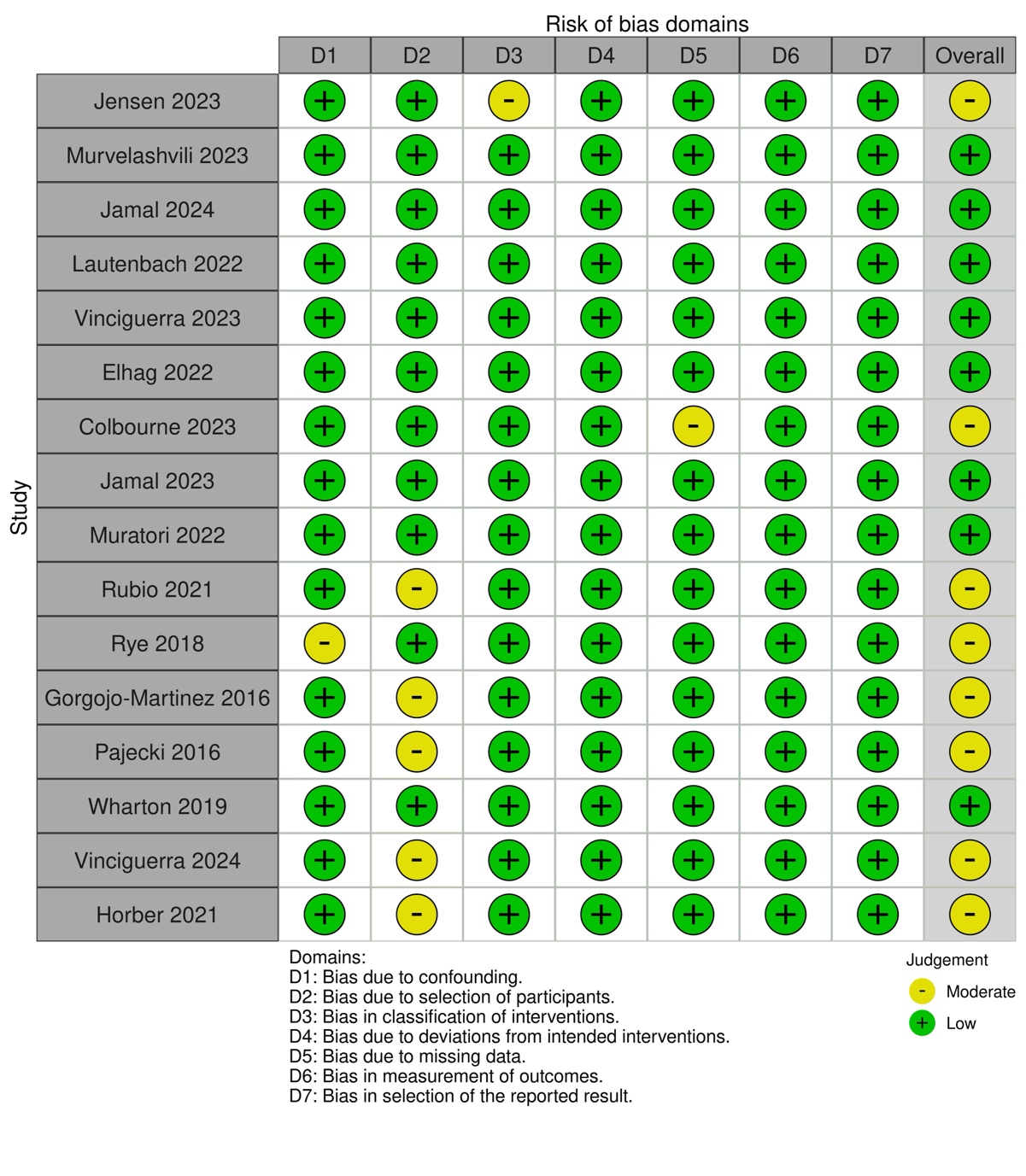


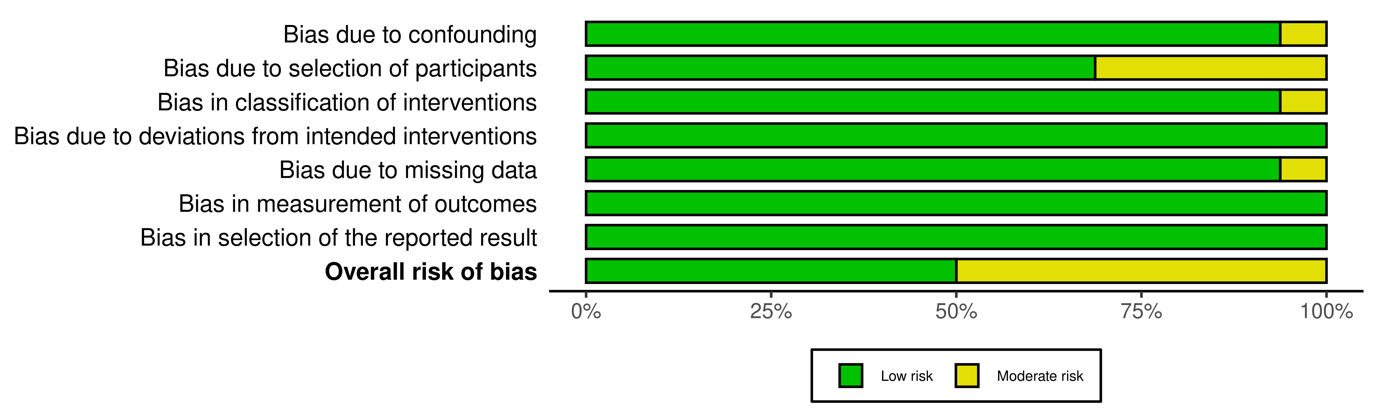


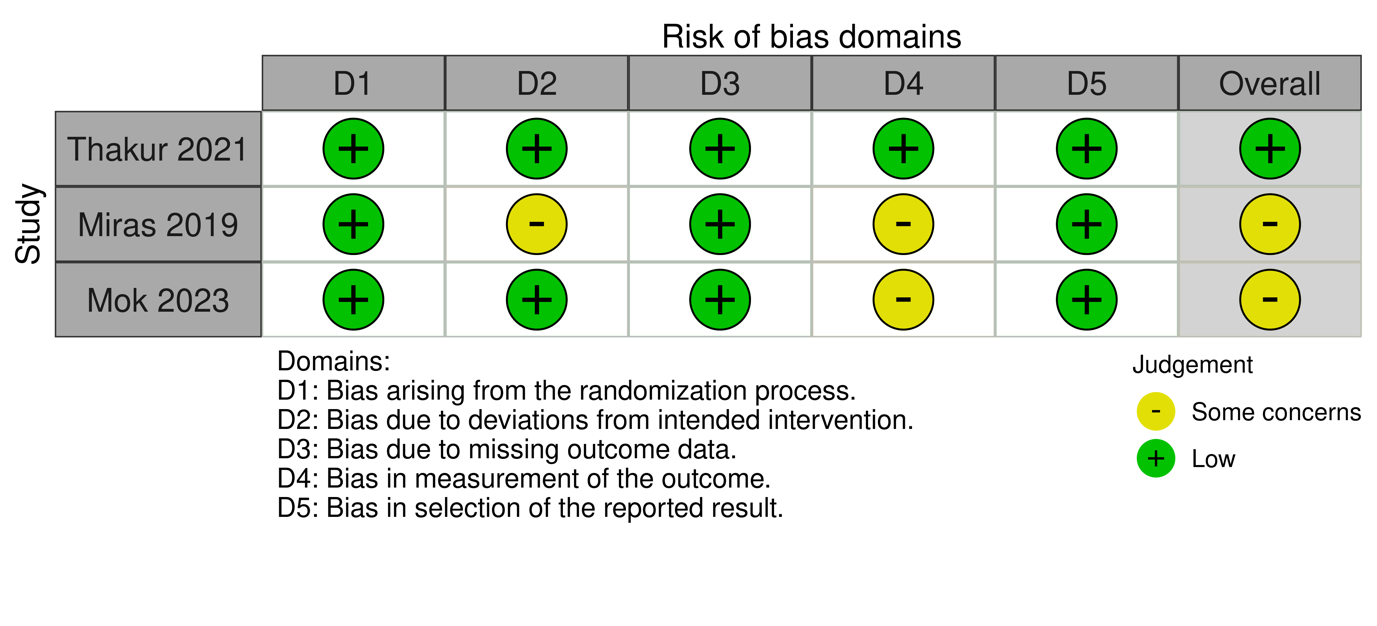

Supplement: Supplementary file 1 — Supplementary Material 1 [file 423_2025_3831_MOESM1_ESM.docx]
