# Supplementary material for: GLP-1 receptor agonists as an adjunct to bariatric surgery for weight loss and metabolic outcome improvement: a systematic review and meta-analysis
Source: Langenbecks Arch Surg. 2025 Oct 10;410(1):295. doi: 10.1007/s00423-025-03831-4 (PMC12513976; doi:10.1007/s00423-025-03831-4)
Supplement: Supplementary file 2 — Supplementary Material 2 [file 423_2025_3831_MOESM2_ESM.docx]

Funnel plot graphs for meta-analysis of primary outcomes

Figure 1A. Any GLP-1 RA- longest follow up- BMI change

Figure 1B. Any GLP-1 RA- longest follow up- weight change

Figure 2A. Liraglutide-longest follow up-BMI change

Figure 2B. Liraglutide-longest follow up-Weight change

Figure 3A. Liraglutide-BMI change from baseline –subgroup analysis

Figure 3B. Liraglutide-Weight change from baseline –subgroup analysis

Figure 4.Liraglutide vs Placebo. Total weight loss

Figure 5. Semaglutide vs Liraglutide. Percentage weight loss
